# Ca^2+^‐permeable AMPA receptors and their auxiliary subunits in synaptic plasticity and disease

**DOI:** 10.1113/JP279029

**Published:** 2021-02-21

**Authors:** Stuart G. Cull‐Candy, Mark Farrant

**Affiliations:** ^1^ Department of Neuroscience, Physiology and Pharmacology University College London Gower Street London WC1E 6BT UK

**Keywords:** AMPA receptors, amyotrophic lateral sclerosis, anoxia, auxiliary subunits, CKAMP44, calcium‐permeable AMPA receptors, cocaine, cornichon, fear conditioning, GluA2, GSG1L, ionotropic glutamate receptors, malignant glioma, neurological disorder, pain, stargazin, synaptic plasticity, synaptic transmission, TARPs

## Abstract

AMPA receptors are tetrameric glutamate‐gated ion channels that mediate a majority of fast excitatory neurotransmission in the brain. They exist as calcium‐impermeable (CI‐) and calcium‐permeable (CP‐) subtypes, the latter of which lacks the GluA2 subunit. CP‐AMPARs display an array of distinctive biophysical and pharmacological properties that allow them to be functionally identified. This has revealed that they play crucial roles in diverse forms of central synaptic plasticity. Here we summarise the functional hallmarks of CP‐AMPARs and describe how these are modified by the presence of auxiliary subunits that have emerged as pivotal regulators of AMPARs. A lasting change in the prevalence of GluA2‐containing AMPARs, and hence in the fraction of CP‐AMPARs, is a feature in many maladaptive forms of synaptic plasticity and neurological disorders. These include modifications of glutamatergic transmission induced by inflammatory pain, fear conditioning, cocaine exposure, and anoxia‐induced damage in neurons and glia. Furthermore, defective RNA editing of GluA2 can cause altered expression of CP‐AMPARs and is implicated in motor neuron damage (amyotrophic lateral sclerosis) and the proliferation of cells in malignant gliomas. A number of the players involved in CP‐AMPAR regulation have been identified, providing useful insight into interventions that may prevent the aberrant CP‐AMPAR expression. Furthermore, recent molecular and pharmacological developments, particularly the discovery of TARP subtype‐selective drugs, offer the exciting potential to modify some of the harmful effects of increased CP‐AMPAR prevalence in a brain region‐specific manner.

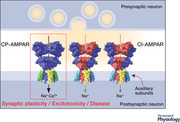

## Graphical abstract

**Abstract figure legend** AMPARs containing GluA2 (red subunits) are Ca^2+^‐impermeable (CI‐AMPARs). Those that lack GluA2 are Ca^2+^‐permeable (CP‐AMPARs) and are implicated in diverse forms of synaptic plasticity and disease. Both native CP‐ and CI‐AMPARs contain various auxiliary subunits (shown as yellow, green or turquoise) that affect AMPAR function and play a role in the regulation of relative CP‐/CI‐AMPAR prevalence. Image based on PDB model 6NJM.

## Introduction

The basic properties of AMPA‐type glutamate receptors (AMPARs) shape many of the key features of fast excitatory transmission in the CNS. Together with NMDA‐type glutamate receptors these ligand‐gated non‐selective cation channels are involved both in synaptic signalling and the induction of various forms of synaptic plasticity (Traynelis *et al*. 2010; Huganir & Nicoll, 2013; Greger *et al*. 2017). At many synapses, AMPAR changes are primarily responsible for the expression of plasticity. Most notably, changes in their number or function underlie the activity‐dependent strengthening or weakening of synaptic contacts, as seen in the processes of long‐term potentiation and depression, the homeostatic adjustments that maintain neuronal excitability, and many other important forms of plasticity (Diering & Huganir, 2018).

Here we give a brief overview of those forms of plasticity that involve a change in the synaptic expression of one particular class of AMPAR – the calcium‐permeable AMPA receptors (CP‐AMPARs). These have emerged as important participants not only in a variety of conventional plasticities, but also in detrimental forms that are implicated in various neurological conditions. Most, if not all AMPARs, are associated with transmembrane auxiliary proteins that influence the receptors’ biogenesis, their post‐ and presynaptic localization at synapses, and their functional properties (Jackson & Nicoll, 2011*b*; Rigby *et al*. 2015; Greger *et al*. 2017; Schwenk *et al*. 2019). We have, therefore, focused our review on the results from studies aimed at identifying specific roles for transmembrane auxiliary proteins in normal and detrimental forms of CP‐AMPAR regulation. As much of the work described here has depended on the identification of CP‐AMPARs from their hallmark properties, we start by summarizing these, and the way in which they are modified when receptors are assembled with particular auxiliary proteins.

### Functional hallmarks of pore‐forming and auxiliary proteins

**Figure 1:**
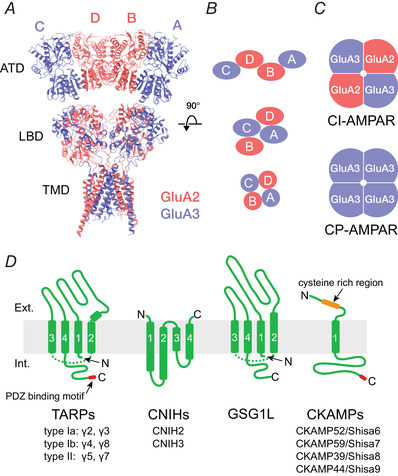
Architecture of AMPARs and key auxiliary subunits *A*, structure of a native heterotetrameric GluA2/3 receptor (PDB 6NJM; Zhao *et al*. 2019) with auxiliary subunits removed, showing the three‐layer arrangement formed from the amino terminal‐, ligand binding‐ and transmembrane domains (ATD, LBD, TMD), with the classical overall ‘Y’‐shape. The GluA2 subunits (positions B and D) are shown in red and the GluA3 subunits (positions A and C) are shown in blue. *B*, positions of the subunits within the ATD, LBD and TMD layers when viewed from the top (extracellular surface) of the receptor, along the overall twofold axis of symmetry. Of note, the arrangement of core subunits in AMPARs is not as strict as seen in NMDARs (Greger *et al*. 2017), and for this native AMPAR the positioning of the GluA2 subunits differs from the A/C positions reported for the first recombinant heteromeric GluA2/3 structure (Herguedas *et al*. 2016). Nevertheless, the fourfold symmetry of the TMD layer is common to both. *C*, cartoon representation of the TMD layer arrangement for a Ca^2+^‐impermeable (CI‐) AMPAR containing Q/R edited GluA2 subunits and a GluA2‐lacking Ca^2+^‐permeable (CP‐) AMPAR. *D*, schematic illustrations of AMPAR key auxiliary subunits. TARPs and GSG1L belong to the claudin superfamily and have four transmembrane α‐helices (numbered) and similar overall structures. Type Ia (γ2, 3) and Type 1b (γ4, 8) TARPs have canonical TTPV PDZ binding motifs whereas Type II TARPs (γ5, 7) have atypical PDZ binding motifs (SSPC and TSPC). Note that because the transmembrane helices form a bundle within the membrane the TM2/TM3 linker (dotted) is shorter than shown. CNIHs also have four transmembrane α‐helices but both the N and C termini are extracellular (Nakagawa, 2019). CKAMPs have a single transmembrane α‐helix, an extracellular cysteine‐rich region (the cysteine knot) and a PDZ binding motif (EVTV).

The properties of AMPARs, notably their kinetics and Ca^2+^ permeability, reflect the nature of the receptors’ constituent subunits (Traynelis *et al*. 2010) and auxiliary subunits (Jackson & Nicoll, 2011, *b*). The main players are depicted in Fig. 1.

Subunit composition can vary across brain regions and between cell types and can change during development and in response to neuronal activity. Of the four homologous pore‐forming subunits (GluA1–GluA4) the GluA2 subunit plays a particularly critical role in determining AMPAR behaviour. GluA2 pre‐mRNA is subject to nucleotide editing (mRNA editing) that results in the conversion of a genetically encoded glutamine (Q) to an arginine (R) at position 607 – the Q/R site in the pore‐forming loop of M2. This switch, from a neutral to a positively charged residue in the channel's ionic selectivity filter, means that unlike GluA2‐lacking AMPARs those containing GluA2 are Ca^2+^‐impermeable (Burnashev *et al*. 1992). Q/R editing within GluA2's pore loop is highly efficient and serves not only to control Ca^2+^ permeability but also to increase the proportion of GluA2‐containing surface receptors by limiting the exit of GluA2 from the endoplasmic reticulum except when associated with unedited subunits (Greger *et al*. 2002; 2003).

**Figure 2:**
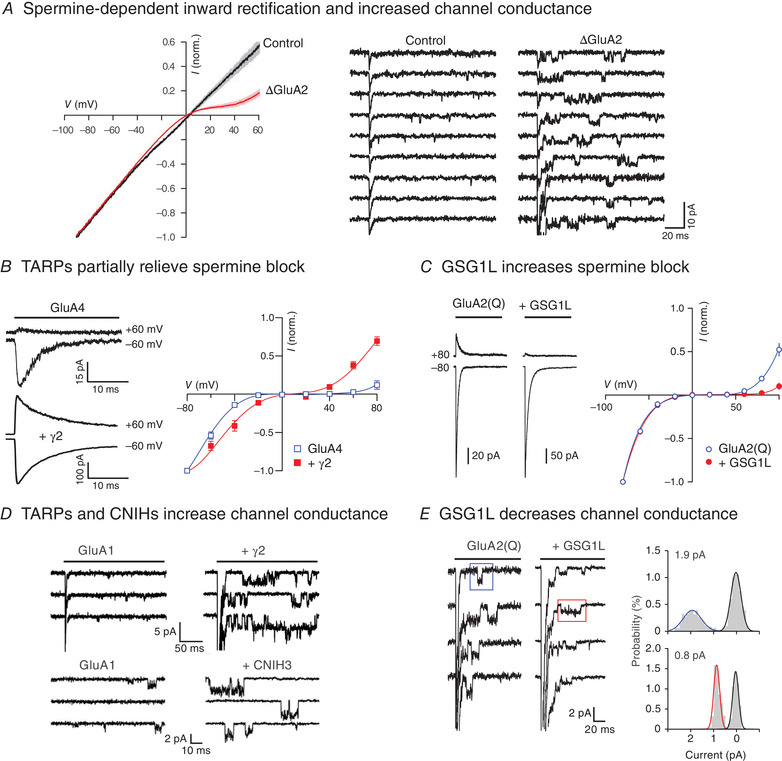
Functional hallmarks of CP‐AMPARs lacking edited GluA2 Selected recordings of native (*A*) and recombinant (*B–E*) CP‐AMPARs. *A*, left, *I‐V* relationships of whole‐cell responses to bath‐applied AMPA (20 μm) recorded from untreated cerebellar granule cells (control) and from cells transfected with short interfering RNAs to disrupt GluA2 production (ΔGluA2). Knockdown of GluA2 promotes spermine‐dependent inward rectification. Right, representative responses from granule cell outside‐out patches to application of AMPA (1 mm, 100 ms, −60 mV). Long‐lived bursts of channel openings are present in the tail of the currents from GluA2 knockdown cells while in control patches only a few smaller and briefer openings are discernible (modified from Studniarczyk *et al*. 2013). *B*, left, representative glutamate‐evoked (100 ms, 10 mm) currents at +60 and −60 mV for homomeric GluA4 AMPARs in the absence or presence of γ2. Right, inwardly rectifying *I‐V* relationships for peak currents, showing reduced rectification in the presence of γ2 (modified from Soto *et al*. 2007). *C*, representative glutamate‐evoked currents (10 mm, 100 ms with 100 μm spermine) and normalized peak *I*‐*V* relationships showing increased rectification in the presence of GSG1L (modified from McGee *et al*. 2015). *D*, top, resolved single‐channel openings at −80 mV in the tail of macroscopic currents (truncated), recorded from homomeric GluA1 AMPARs expressed in the absence and presence of γ2, illustrating the increased single‐channel conductance, increased open probability and slowed kinetics in the presence of TARP (modified from Coombs & Cull‐Candy, 2009). Bottom, representative single‐channel currents recorded in outside‐out patches from tsA201 cells with GluA1 expressed alone or with CNIH3 (−80 mV; 10 mm glutamate) (modified from Coombs *et al*. 2012). *E*, resolved single‐channel openings at −80 mV in the tail of macroscopic currents (truncated) from homomeric unedited GluA2(Q) AMPARs, with all‐point amplitude histograms from individual channel events (modified from McGee *et al*. 2015).

As shown in Fig. 2, CP‐AMPARs – those lacking an edited GluA2 subunit – have a higher single‐channel conductance than the GluA2‐containing calcium‐impermeable receptors (CI‐AMPARs) (Swanson *et al*. 1997; Feldmeyer *et al*. 1999). Thus, directly resolved channel openings and estimates of single‐channel conductance, obtained using non‐stationary fluctuation analysis (NSFA) of miniature excitatory postsynaptic currents (mEPSCs) or macroscopic currents activated by fast application of glutamate onto outside‐out patches (see Traynelis *et al*. 1993; Soto *et al*. 2007), can provide a valuable clue to the presence of CP‐AMPARs. Additionally, currents mediated by CP‐AMPARs are blocked at depolarized membrane potentials by the endogenous intracellular polyamines spermine and spermidine, giving rise to inwardly or bi‐rectifying current‐voltage relationships (Bowie & Mayer, 1995; Kamboj *et al*. 1995; Koh *et al*. 1995). CP‐AMPAR channels are also susceptible to selective use‐dependent block from the outside by a variety of exogenous molecules, including the polyamine wasp toxin philanthotoxin‐4,3,3 (PhTx‐433; Washburn & Dingledine, 1996), the Joro spider toxin analogue 1‐naphthylacetyl spermine (NASPM; Tsubokawa *et al*. 1995) and the adamantane derivative IEM‐1460 (Magazanik *et al*. 1997). These three characteristics, together with others given in Table 1, have been widely used to identify changes in CP‐AMPAR prevalence linked to synaptic plasticity (Liu & Cull‐Candy, 2000; Gardner *et al*. 2005; Plant *et al*. 2006; Lamsa *et al*. 2007; Sanderson *et al*. 2016; Park *et al*. 2019; Purkey & Dell'Acqua, 2020) and disease (Liu *et al*. 2004; Noh *et al*. 2005; Quintana *et al*. 2015; Bellone & Luscher, 2006; Conrad *et al*. 2008; Scheyer *et al*. 2018; Adotevi *et al*. 2020).

**Table 1:** Functional hallmarks of CP‐AMPARs, and their modification by auxiliary subunits

Property	References
**Higher single‐channel conductance than CI‐AMPARs**	Swanson *et al*. 1997
mean conductance increased (>30 pS) by TARPs and CNIHs	Tomita *et al*. 2005; Soto *et al*. 2009; Coombs *et al*. 2012
resolvable opening of singly‐liganded TARPed receptors	Coombs *et al*. 2017
mean conductance decreased (<15 pS) by GSG1L	McGee *et al*. 2015
**Inwardly rectifying *I‐V* relationships**	
due to block by endogenous intracellular polyamines	Bowie & Mayer, 1995; Kamboj *et al*. 1995; Koh *et al*. 1995
block partially relieved by TARPs and CNIHs	Soto *et al*. 2007; Coombs *et al*. 2012; Brown *et al*. 2018
block increased by GSG1L	McGee *et al*. 2015
**Block by exogenous extracellular organic cations** PhTx‐433 (block enhanced by TARPs) NASPM IEM‐1460	Washburn & Dingledine, 1996; Jackson *et al*. 2011 Tsubokawa *et al*. 1995 Magazanik *et al*. 1997

In addition to the pore‐forming subunits, proteins belonging to several distinct families have emerged as important AMPAR constituents (Fig. 1), acting as auxiliary subunits that influence the receptors’ biogenesis and localization within the cell membrane, as well as their biophysical and pharmacological properties. Those that contribute to the proteomic ‘core’ of the receptor (Schwenk *et al*. 2012) include the transmembrane AMPAR regulatory proteins (TARPs; γ2, ‐3, ‐4, ‐5, ‐7 and ‐8) (Jackson & Nicoll, 2011*b*), two widely occurring members of the cornichon family (CNIH2 and ‐3) (Schwenk *et al*. 2009; Nakagawa, 2019), and the germ cell‐specific gene 1‐like protein (GSG1L) (Schwenk *et al*. 2014; Shanks *et al*. 2014). Other protein families that contribute to the ‘peripheral’ components of the proteome include the cysteine‐knot AMPAR modulating proteins (CKAMP52, ‐59, ‐39 and ‐44; Shisa6, ‐7, ‐8 and ‐9) (Jacobi & von Engelhardt, 2017; 2021), two proline‐rich transmembrane proteins (PRRT1 and ‐2) (Schwenk *et al*. 2012, 2014; Shanks *et al*. 2014; Matt *et al*. 2018), and the leucine‐rich repeat transmembrane neuronal protein 4 (LRRTM4) (Schwenk *et al*. 2014). Recent work has also shown that different auxiliary proteins associate with the AMPAR subunits during their assembly within the endoplasmic reticulum (ER). FRRS1l (ferric chelate reductase 1 like) protein, in complex with CPT1c (carnitine *O*‐palmitoyl‐transferase 1c), mediate the formation of GluA tetramers from monomers initially associated with ABHD6 (α/β‐hydrolase domain‐containing 6), and allow their co‐assembly with the core AMPAR auxiliary subunits in readiness for exit from the ER and subsequent insertion in the plasma membrane (Schwenk *et al*. 2019).

Many of the auxiliary subunits have been shown to modify basic properties of both CI‐ and CP‐ forms of AMPARs (see Table 1). Thus, the TARPs typically increase single‐channel conductance, slow the channel kinetics, alter the pharmacology of agonists, antagonists and allosteric modulators, and enhance receptor trafficking to the cell surface (Jackson & Nicoll, 2011*b*; Greger *et al*. 2017; Jacobi & von Engelhardt, 2021). The degree to which they influence the AMPAR properties varies between TARP sub‐family members. For example, type Ib TARPs (γ4 and γ8) slow the channel kinetics and can increase single‐channel conductance to a greater extent than type Ia (γ2 and γ3) or type II (γ5 and γ7) TARPs (Cho *et al*. 2007; Milstein *et al*. 2007; Kato *et al*. 2010; Jackson *et al*. 2011).

In the case of GluA2‐lacking CP‐AMPAR, co‐assembly with TARPs or CNIHs increases their already high (relative to CI‐AMPARs) single‐channel conductance (Tomita *et al*. 2005; Soto *et al*. 2009; Coombs *et al*. 2012) (see Fig. 2). For homomeric GluA4 receptors, it is thought that this increase reflects an enhanced proportion of events opening to their higher sub‐conductance states (Tomita *et al*. 2005). By contrast, for GluA1 receptors there appears to be an increase in the absolute amplitude of the maximum conductance state (Shelley *et al*. 2012). In all cases, the unusually high single‐channel conductance of TARP associated CP‐AMPARs is often sufficient to allow these to be distinguished from the TARPed CI‐AMPARs, or indeed from TARPless AMPARs (see Bats *et al*. 2012). CNIHs and TARPs increase conductance to a similar extent (Coombs *et al*. 2012), while CKAMPs/Shisas produce only a marginal increase in channel conductance (Jacobi & von Engelhardt, 2017). In striking contrast with the other core auxiliary subunits, GSG1L reduces both the weighted mean single‐channel conductance (by ∼50%) and the calcium permeability of CP‐AMPARs, while increasing the channel's polyamine‐dependent rectification (Fig. 2). Thus, increased expression of GSG1L has been found to reduce EPSC amplitude (McGee *et al*. 2015; Gu *et al*. 2016).

Co‐assembly of CP‐AMPARs with TARPs or CNIHs partially relieves the block by intracellular polyamines (Cho *et al*. 2007; Soto *et al*. 2007; Coombs *et al*. 2012; Brown *et al*. 2018), by increasing polyamine permeation (Brown *et al*. 2018). By contrast, TARPs enhance CP‐AMPAR block by extracellular polyamine toxin PhTx‐433 (Jackson *et al*. 2011). This block is more effective when the receptors are activated by the full agonist glutamate rather than by the partial agonist kainate, suggesting that the block is favoured when the channels open predominantly to higher conductances (Jackson *et al*. 2011). Indeed, the degree of block of CI‐AMPARs by extracellular PhTx‐74, a related polyamine toxin, is positively correlated with their single‐channel conductance (Jackson *et al*. 2011). While a detailed mechanism for this observation is lacking, the idea that TARP‐increased channel conductance and altered polyamine block might originate from a simple increase in the pore size can be excluded, as functional evidence suggests that the CP‐AMPAR channel pore diameter is unaltered by TARPs (Soto *et al*. 2014).

Recent cryo‐EM work has solved the structures of γ2‐associated CP‐AMPARs (homomeric unedited GluA2) in the presence of the exogenous channel blockers NASPM, IEM‐1460 and argiotoxin‐636 (Twomey *et al*. 2018). Each blocking molecule sits along the pore axis of the channel with its hydrophobic head below the channel's gate and above the selectivity filter. The hydrophobic head stops the molecule from readily permeating through the channel, and the tail extends down through the selectivity filter. For all three blocking molecules the channel's Q/R site glutamines, which form the narrowing constriction of the pore, appear to be the main anchoring point for their tail. It is therefore suggested that the blockers supress current flow by plugging the ion channel, without interfering with the gating mechanism (Twomey *et al*. 2018). For intracellular polyamines, in addition to the Q/R site, electronegative charge provided by an aspartate residue at the ‘Q/R +4’ site is a key determinant of block. Neutralization of this charge decreases spermine block (Panchenko *et al*. 1999; Soto *et al*. 2014) as well as reducing channel conductance (Soto *et al*. 2014).

Co‐assembly with TARP family members produces another surprising change in AMPAR pharmacology, transforming the competitive antagonist CNQX into a partial agonist, and increasing the efficacy of the partial agonist kainate (Jackson & Nicoll, 2011, *b*). Of note, not all TARPs render AMPARs sensitive to activation by CNQX. The type II TARP γ7 is ineffective in this respect (Bats *et al*. 2012), although it is still capable of relieving intracellular polyamine block and increasing channel conductance. Interestingly, CNIHs fail to convert CNQX to a partial agonist, and only marginally increase the efficacy of the partial agonist kainate (Shi *et al*. 2010).

TARPs also reduce AMPAR desensitization and enhance the efficacy of glutamate at the concentrations that prevail during fast transmission (Cho *et al*. 2007; Milstein *et al*. 2007; Ben‐Yaacov *et al*. 2017; Coombs *et al*. 2017). Recently, we examined the influence of TARPs on AMPARs gated by low concentrations of glutamate in order to obtain information about receptor activation during slower and more diffuse synaptic events. By first saturating the receptors with the antagonist NBQX, then rapidly switching into glutamate, it was possible to observe directly the sequential gating responses as individual molecules of the competitive blocker slowly unbound to be replaced by glutamate. This provided information about the time course of channel activation (over hundreds of milliseconds) and revealed the sub‐conductance level associated with each occupancy state of an individual TARPed receptor. Unlike TARPless receptors examined under similar conditions, that were found to exhibit three open levels, for TARPed CP‐AMPARs, four directly resolved conductance steps were evident during the channel activation process. This indicates an enhancement of glutamate efficacy such that even singly liganded receptors are able to generate channel openings. While the single‐channel conductance of such events is relatively small, ∼10% of the fully open state (Coombs *et al*. 2017), the overall effect of TARPs on glutamate efficacy will facilitate synaptic signalling and Ca^2+^ influx (for CP‐AMPARs) during prolonged exposure to low transmitter concentrations. This is likely to enhance AMPAR responses such as those that occur during synaptic spillover and delayed clearance of transmitter (DiGregorio *et al*. 2007; Zampini *et al*. 2016).

### Normal and maladaptive forms of plasticity involving CP‐AMPARs

A rapid and lasting change in the prevalence of GluA2‐containing AMPARs, and thus in the fraction of CP‐AMPARs, is a key feature of many different forms of synaptic plasticity (see Table 2). We will briefly summarize some of these, before focusing (in the section on: Auxiliary subunits implicated in native CP‐AMPAR regulation) on those forms where information is available about the involvement of auxiliary subunits in this regulation.

**Table 2:** Conventional and detrimental forms of plasticity involving CP‐AMPAR changes

Condition/trigger	Brain region	AMPAR change	References
High frequency activity‐induced LTD	Cerebellum – granule cell‐stellate cell synapse	CP‐ to CI‐AMPARs	Liu & Cull‐Candy, 2000; Gardner *et al*. 2005; Kelly *et al*. 2009
LTP	Hippocampal CA1 synapses	Transient CP‐AMPAR incorporation	Plant *et al*. 2006; Lu *et al*. 2007; Guire *et al*. 2008; Park *et al*. 2019 (but see Adesnik & Nicoll, 2007; Gray *et al*. 2007; Granger *et al*. 2013)
LTD	Hippocampal CA1 synapses	Transient CP‐AMPAR incorporation	Sanderson *et al*. 2016
Homeostatic plasticity	Hippocampal CA1 synapses	Transient CP‐AMPAR incorporation	Sutton *et al*. 2006; Sanderson *et al*. 2018
Inflammatory pain	Spinal cord – superficial dorsal horn lamina II neurons	CI‐ to CP‐AMPARs	Kopach *et al*. 2011; Sullivan *et al*. 2017
Fear conditioning and fear extinction	Cerebellum; lateral amygdala	CP‐ to CI‐AMPARs	Clem & Huganir, 2010; Liu *et al*. 2010
Anoxia (stroke)	Hippocampus – CA1 pyramidal cells	CI‐ to CP‐AMPARs	Noh *et al*. 2005; Quintana *et al*. 2015
Anoxic damage in oligodendrocytes	Various	CI‐ to CP‐AMPARs	Follett *et al*. 2004; Zonouzi *et al*. 2011; Ceprian & Fulton, 2019
Cocaine exposure	Nucleus accumbens; Ventral tegmental area	CI‐ to CP‐AMPARs	Bellone & Luscher, 2006; Selvakumar *et al*. 2014
Prion protein mutations	Spinal cord – superficial dorsal horn lamina II neurons	CI‐ to CP‐AMPARs	Ghirardini *et al*. 2020
Glaucoma	Retina – ganglion cells	CI‐ to CP‐AMPARs	Sladek & Nawy, 2020
GluA2 editing defects (Alzheimer's disease, ALS, seizure vulnerability, malignant gliomas)	Various	CI‐ to CP‐AMPARs	Maas *et al*. 2001; Gaisler‐Salomon *et al*. 2014; Yamashita & Kwak, 2019; Konen *et al*. 2020

**Figure 3:**
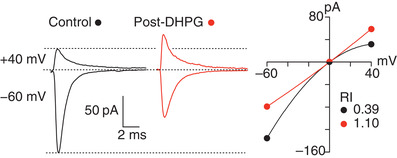
An example of CP‐AMPAR plasticity Application of the group 1 mGluR agonist (*S*)‐3,5‐dihydroxyphenylglycine (DHPG) induces a persistent synaptic depression and change in the rectification of EPSCs recorded from stellate cells in acute slices of mouse cerebellum. Left, averaged parallel fibre‐evoked EPSCs recorded at −60 and +40 mV before and after the application of 50 μm DHPG (10 min). Dashed lines indicate baseline and peak currents for control EPSCs. Right, corresponding *I‐V* relationships. Control EPSCs show inward rectification, with a rectification index (RI) of 0.39, for this example. Following DHPG application the *I‐V* relationship became linear (RI of 1.1), indicating a shift from CP‐ to CI‐AMPARs. A similar shift can be induced by synaptic activation of mGluRs. (modified from Kelly *et al*. 2009).

As first described at cerebellar parallel fibre‐to‐stellate cell synapses, where a proportion of synaptic AMPARs are calcium‐permeable, high frequency activity can trigger a change in the current‐voltage (*I‐V*) relationship of the EPSCs. This effect can be replicated by activation of synaptically located metabotropic (mGluR1) receptors (Fig. 3). Decreased CP‐AMPAR expression following activation of mGluR1s is a theme in several different neuron types and of particular interest given its relevance to drug addiction (see below). The rapid alteration from inwardly rectifying to linear *I‐V* is accompanied by a reduction in EPSC amplitude (at negative potentials), reflecting the replacement of the CP‐AMPARs by lower conductance CI‐AMPARs (Liu & Cull‐Candy, 2000; Gardner *et al*. 2005; Kelly *et al*. 2009). The activation of both CP‐AMPARs and mGluR1/5 is necessary to trigger a rise in intracellular Ca^2+^ required for this AMPAR plasticity, implying the presence of a self‐regulating mechanism (Kelly *et al*. 2009; Liu *et al*. 2010; Bats et al. 2012; 2013). Conversely, plasticity involving a lasting increase in CP‐AMPAR expression appears to underlie several forms of synaptic remodelling that are physiologically and behaviourally important. These include postsynaptic changes in lamina ll spinal cord neurons as a result of inflammatory pain (Kopach *et al*. 2011; Sullivan *et al*. 2017) and synaptic remodelling associated with fear conditioning and fear extinction (Clem & Huganir 2010; Liu *et al*. 2010).

Many detrimental types of plasticity have been described that involve an increase in CP‐AMPAR expression. These include the cocaine‐induced modification of glutamatergic transmission onto dopamine neurons in the ventral tegmental area and nucleus accumbens (Bellone & Luscher, 2006; Selvakumar *et al*. 2014), anoxia‐induced decreases in GluA2 expression in hippocampal CA1 cells (Noh *et al*. 2005; Quintana *et al*. 2015), and increased CP‐AMPAR expression in oligodendrocyte lineage cells that can follow hypoxia during gestation (Follett *et al*. 2004; Zonouzi *et al*. 2011; Ceprian & Fulton, 2019). Additionally, certain mutations in prion proteins can result in disorders that involve excitotoxic neurodegeneration caused by increased expression of neuronal CP‐AMPARs (Ghirardini *et al*. 2020). And in a mouse model of glaucoma, elevated intraocular pressure causes an increase in damaging CP‐AMPAR expression in specific subpopulations of retinal ganglion cells (Sladek & Nawy, 2020).

Although not a conventional plasticity, it is also interesting to note that the aberrant expression of CP‐AMPARs can result from the downregulation of mRNA editing at the Q/R site of GluA2 (Wright & Vissel, 2012; Slotkin & Nishikura, 2013). This has been suggested to play a role in Alzheimer's disease (Gaisler‐Salomon *et al*. 2014), in both sporadic and familial amyotrophic lateral sclerosis (motor neuron disease) (Yamashita & Kwak, 2019), in seizure vulnerability (Konen *et al*. 2020), and in the growth of malignant gliomas (Maas *et al*. 2001).

### Auxiliary subunits implicated in native CP‐AMPAR regulation

As various auxiliary proteins, including TARPs, CNIHs and GSG1L, can modify the biophysical behaviour and pharmacology of both CP‐ and CI‐recombinant AMPARs, the question arises, are there specific auxiliary proteins that selectively regulate the trafficking and localization of native CP‐AMPARs? To date, the auxiliary subunits involved in CP‐AMPAR regulation and plasticity have been examined in only a relatively small number of cell types, but these studies have started to throw some light on this issue (see Table 3).

**Table 3:** Auxiliary subunits implicated in CP‐AMPAR regulation

Cell type/synapses	Brain region	Auxiliary subunits	References
Bergmann glia	Cerebellum	γ5, γ7	Fukaya *et al*. 2005; Soto *et al*. 2009; Yamazaki *et al*. 2010
Oligodendrocyte precursor cells	Cerebellum; optic nerve	γ2, CNIH2/3	Zonouzi *et al*. 2011; Coombs *et al*. 2012
Gliomas	Various	CPT1c	Chen *et al*. 2020
CA1 pyramidal cells	Hippocampus	γ2, γ8, CNIH2, GSG1L	Matsuda *et al*. 2013; Schwenk *et al*. 2014; Park *et al*. 2016; Sheng *et al*. 2018
Medium spiny neurons	Nucleus accumbens	γ2, γ4	Ferrario *et al*. 2011
Lamina II neurons	Spinal cord superficial dorsal horn	γ2, γ8	Sullivan *et al*. 2017
Granule cell‐stellate cell synapses	Cerebellum	γ7, γ2	Bats *et al*. 2012; Studniarczyk *et al*. 2013; Yamazaki *et al*. 2015

[Correction made on 3 March 2021, after first online publication: The table has been updated to correct the header in the third column from ‘AMPAR change’ to ‘Auxiliary subunits’.]

#### CP‐AMPARs in glia, oligodendrocyte precursor cells and glioma

Unlike most neurons Bergmann glia (BG), the main radial glia within the cerebellum, appear to be entirely devoid of GluA2 and thus express exclusively CP‐AMPARs. The activation of these receptors by glutamate is crucial for BG cell processes to correctly ensheath the synapses present on Purkinje cell dendritic spines, thereby enabling fast transmission, transmitter removal, and optimal synaptic integration (Iino *et al*. 2001; Saab *et al*. 2012). BG strongly express γ5, a TARP that is absent from all other cerebellar cells (Fukaya *et al*. 2005). The CP‐AMPAR‐mediated quantal events underlying neuron‐glia signalling in BG display single‐channel and kinetic properties indicative of γ5‐associated receptors, and there is good evidence to suggest the receptors are assembled from GluA1/γ5 and/or GluA4/γ5 (Soto *et al*. 2009). However, it is notable that BG also express γ7 (Yamazaki *et al*. 2010). It thus remains possible that the functional properties of CP‐AMPARs in BG cells are regulated by both γ5 and γ7. It is of particular interest that BG, which are unusual in expressing only CP‐AMPARs, express only type II TARPs. This strongly suggests that, in some cell types at least, type II TARPs are sufficient to deliver CP‐AMPARs to the plasma membrane.

CP‐AMPARs also play an important role in oligodendrocyte precursor cell (OPC) proliferation, differentiation, migration and neuron‐glial signalling (Harlow *et al*. 2015; Chen *et al*. 2018). However, they also render OPCs particularly susceptible to damage during gestation and early stages of development (Volpe, 2009). We have identified several factors that regulate the AMPAR subtypes present in OPCs. Notably, activation of group 1 mGluRs in these cells triggers an increase in the proportion of CP‐AMPARs, signified by an increase in inward rectification of glutamate‐evoked currents. Furthermore, the kinetic features and underlying channel conductance of the CP‐AMPAR‐mediated currents suggest that these are TARP‐associated AMPARs. Oligodendrocyte lineage cells express predominantly GluA2, ‐3 and ‐4 subunits, although GluA3 and ‐4 may predominate (Zonouzi *et al*. 2011; Zhang *et al*. 2014). Of these, GluA4 is thought to be particularly important in generating excitotoxic damage (Begum *et al*. 2018). TARPs γ2, ‐3, ‐4 and ‐5 have all been detected in OPCs using RT‐PCR (Zonouzi *et al*. 2011). In addition, antibody labelling in these cells has verified the presence of TARPs that contain the TTPV motif at their C‐ terminus (Zonouzi *et al*. 2011), indicating that the predominant forms present are γ2, ‐3 or ‐4 (rather than γ5). Transfection of OPCs with a form of γ2 that lacked its last 16 residues (including the TTPV motif required for binding to PDZ domain‐containing proteins) was able to inhibit the mGluR‐mediated increase in CP‐AMPARs, leaving a glutamate‐evoked current that was mediated entirely by CI‐AMPARs. This confirmed the importance of type I TARPs in delivery of CP‐AMPARs in these cells (Zonouzi *et al*. 2011). Interestingly, while there is also evidence that CNIHs are associated with AMPARs in OPCs (Coombs *et al*. 2012), in contrast with the TARPs there is no evidence to suggest CNIHs selectively target CP‐AMPARs in OPCs. Thus, γ2 appears to be the primary candidate for CP‐AMPAR trafficking and localization in the OPC plasma membrane. AMPAR signalling is crucial for myelination but seems to enhance oligodendrocyte survival rather than promote myelination itself (Kougioumtzidou *et al*. 2017). Furthermore, OPC proliferation and differentiation are promoted differently, depending on the subunit composition of the AMPARs that are activated by axonal glutamate. OPC proliferation is triggered by expression of unedited CP‐AMPARs, whereas the presence of GluA2‐containing receptors appears to be required for OPCs to respond to differentiation cues (Chen *et al*. 2018). Clearly, understanding the mechanism by which TARPs (probably γ2) target different AMPAR subtypes to influence OPC proliferation and survival could be of considerable therapeutic value.

Gliomas (oligodendrogial or astrocytic primary brain tumours) strongly express CP‐AMPARs. These receptors assemble primarily from GluA1, ‐2 and ‐4. However, the presence of editing deficient forms of GluA2 within glioma cells (Maas *et al*. 2001; Venkataramani *et al*. 2019; Venkatesh *et al*. 2019) means that a high proportion of the AMPARs are highly Ca^2+^ permeable despite the incorporation of GluA2. It has recently been shown that CP‐AMPARs are present at synapses that form between neurons and glioma cells within the tumour, and that their activation promotes tumour cell proliferation and invasiveness. Suppression of activation by genetically perturbing AMPAR signalling with a dominant negative AMPAR subunit, or by the use of AMPAR antagonists such as parampanel (Venkataramani *et al*. 2019; Venkatesh *et al*. 2019) greatly reduces cell proliferation – a feature that has clear therapeutic potential. While little is currently known about the core auxiliary subunits involved in delivery of CP‐AMPARs at these neuron‐glioma synapses, it has long been known that the AMPAR‐associated protein CPT1c is common in gliomas and in a surprisingly wide variety of other cancer cell types. These include lung, breast and pancreatic cells (reviewed in Chen *et al*. 2020). Several recent studies have highlighted the importance of CPT1c in AMPAR biogenesis and shown that it forms an integral part of the AMPAR complex in healthy cells throughout the CNS (Schwenk *et al*. 2012). While it clearly behaves as an interacting protein in heterologous expression systems (Gratacòs‐Batlle *et al*. 2015) it does not appear to modify the functional properties of AMPARs. Within neurons it does not associate with the AMPARs present in the plasma membrane, rather it forms part of the AMPAR assembly within the ER membrane, where it is crucial in tetramerization of the receptor dimers (Schwenk *et al*. 2019). Thus, for reasons that are far from clear, many tumour cell types including ones not associated with the nervous system, express AMPARs (and hence CPT1c) that appear to play a role in cell proliferation. This has been utilised as a novel marker of cancer cells as well as a potential therapeutic target that can be supressed (Zhang *et al*. 2017).

#### Acidosis/hypoxia in hippocampal CA1 region

Pyramidal cells in the CA1 region of the hippocampus are susceptible to damage following ischaemic stroke, where oxygen/glucose deprivation (OGD) promotes excessive glutamate release and acidosis that causes Ca^2+^ influx. This triggers various downstream effects, including an increase in CP‐AMPARs, activation of which allows a further rise in intracellular Ca^2+^ that contributes to the delayed neuronal death (Opitz *et al*. 2000; Noh *et al*. 2005; Quintana *et al*. 2015). The shift in AMPAR subtype involves the rapid and selective endocytosis and lysosomal degradation of GluA2/GluA3 heteromers, a down‐regulation of GluA2 transcription, and the recruitment of extrasynaptic CP‐AMPARs (GluA1/GluA3 or homomeric GluA1) (Koszegi *et al*. 2017).

Of note, a transient recruitment of GluA1‐containing CP‐AMPARs to CA1 synapses has also been proposed to play a role during conventional long‐term potentiation (LTP) and long‐term depression (LTD) (Plant *et al*. 2006; Lu *et al*. 2007; Guire *et al*. 2008; Sanderson *et al*. 2016; Park *et al*. 2019). However, with regard to LTP, there is also evidence against recruitment of CP‐AMPARs (Adesnik & Nicoll, 2007; Grey *et al*. 2007; Granger *et al*. 2013), and the topic remains unresolved (Purkey & Dell'Acqua, 2020). Roles for γ8 and γ2 have been proposed in LTP and LTD at CA1 synapses (Matsuda *et al*. 2013; Park *et al*. 2016; Sheng *et al*. 2018), but their interaction with CP‐AMPAR subtypes has not been examined. Likewise, there is no clear indication of which auxiliary subunits are involved in delivery of CP‐AMPARs following anoxia in CA1. TARPs γ2 and γ8, GSG1L, CNIH2 and CNIH3 are all present, and thus all are potential candidates. Interestingly, GSG1L has been shown to supress CP‐AMPAR function and ‘negatively regulate’ synaptic transmission. Hence, GSG1L attenuates single‐channel conductance and calcium permeability of homomeric AMPARs but increases block by intracellular spermine and increases mEPSC rectification in cultured cerebellar neurons (McGee *et al*. 2015). On the other hand, in hippocampal pyramidal cells knockdown or knockout of GSG1L enhances AMPAR‐mediated synaptic transmission (McGee *et al*. 2015; Gu *et al*. 2016) and enhances LTP at the Schaffer‐collateral pathway (Gu *et al*. 2016).

#### Addictive drug‐induced changes in the ventral tegmental area and nucleus accumbens

Exposure to drugs of abuse causes various forms of synaptic plasticity within brain regions implicated in reward and motivation, notably the nucleus accumbens (NAc) and ventral tegmental area (VTA) (Luscher, 2016; Wolf, 2016). In dopamine neurons of the VTA that project to the NAc, a single exposure to cocaine, for example, alters excitatory transmission by promoting insertion of GluN3A‐containing NMDARs triggering a subsequent switch from CI‐ to CP‐AMPARs and consequent potentiation of the synaptic currents (Bellone & Luscher, 2006; Yuan *et al*. 2013). An increase in the prevalence of CP‐AMPARs is also seen in medium‐spiny neurons of the NAc shell following withdrawal from cocaine (Conrad *et al*. 2008; Scheyer *et al*. 2018). In both cases, the increased neuronal excitation is thought to contribute to enhanced drug‐related behaviours. While there is little information about the identity of auxiliary proteins involved in AMPAR changes in the VTA, biochemical studies using subcellular fractionation and antibody labelling in the NAc have suggested that the newly inserted synaptic GluA1‐containing CP‐AMPARs are associated with γ2, and the extrasynaptic CP‐AMPARs with γ4 (Ferrario *et al*. 2011). It is interesting to note that γ8 is also very abundant in NAc, while γ7 and GSG1L are also present at a lower level (Schwenk *et al*. 2014, Supplementary Table). The involvement of these other potentially relevant auxiliary subunits is unknown. Subsequent studies have revealed an increase in both γ2 and γ4 in NAc following sensitization and withdrawal, and concluded that NMDAR‐driven *S*‐nitrosylation of γ2, which increases GluA1/γ2 association (Selvakumar *et al*. 2009), is necessary for the upregulation of surface GluA1‐containing AMPARs (Selvakumar *et al*. 2014). Interestingly, in animals that have undergone incubation of cocaine craving, activation of mGlu1 receptors in the NAc triggers the endocytosis of the newly inserted CP‐AMPARs (McCutcheon *et al*. 2011). As mGluR1 activation can also drive the synapse to its pre‐drug state in VTA neurons, this lasting change has been suggested to offer a potential therapeutic target for reducing cue‐induced craving (Bellone & Luscher, 2006; Scheyer *et al*. 2018).

#### Hyperalgesia in lamina ll of spinal cord

TARPs γ2 and γ8 are both present in lamina II of the superficial dorsal horn (SDH) of the spinal cord (Sullivan *et al*. 2017), an area involved in nociception. Heightened pain sensitivity associated with peripheral inflammation involves an increase in neuronal excitability and CP‐AMPAR prevalence (Katano *et al*. 2008; Park *et al*. 2009). We have established that one of the mechanisms contributing to peripheral inflammation‐associated changes is synaptic remodelling, characterised by an increase in CP‐AMPARs specifically at the pain fibre synapses (Sullivan *et al*. 2017). Prior to hyperalgesia, transmission from local inputs onto lamina II neurons is mediated by γ2‐associated CI‐AMPARs, while at peripheral pain fibre (C‐fibre) synapses on the same cells it is mediated by CI‐AMPARs associated with a different auxiliary subunit (possibly γ8). The view that γ2 is ‘synapse specific’ and absent from normal C‐fibre synapses prior to hyperalgesia is supported by evidence from immunohistochemical co‐labelling. Interestingly, the inflammation induced remodelling of C‐fibre synapses entails replacement of the γ2‐lacking CI‐AMPARs with γ2‐containing CP‐AMPARs – a change that predictably is lost in the *stargazer* mouse (Sullivan *et al*. 2017), a mutant devoid of functional γ2.

Recent work has identified a number of AMPAR antagonists that are highly selective for γ8‐associated receptors (Kato *et al*. 2016; Maher *et al*. 2016; see below). One of these, LY3130481, has been shown to supress excitatory postsynaptic transmission and attenuate short‐term synaptic plasticity in spinal sensory neurons, and supress behaviour associated with pain perception (Knopp *et al*. 2019). Although the precise role of γ8 in spinal cord pain pathways is still uncertain, this evidence suggests that γ8‐selective antagonists could offer novel therapies for conditions involving chronic pain. Of note, LY3130481 only partially supresses EPSPs in dorsal horn spinal cord neurons, in contrast with the full block produced by the non‐selective AMPAR antagonist GYKI53784. This may reflect the degree of γ8 expression but also the relative expression of other TARPs, specifically γ2. When tested against recombinant receptors, the potency and efficacy of LY3130481 is decreased by co‐expression of γ2 (or γ3) with γ8 (Knopp *et al*. 2019). The precise interplay of γ8 and γ2 in spinal nociceptive signalling remains to be determined. One possibility from earlier work (Sullivan *et al*. 2017) is that γ8, along with γ2, is required for the translocation of CP‐AMPAR at the C‐fibre synapses following peripheral inflammation. In which case, pharmacological block of γ8‐associated receptors could provide a promising approach for suppressing the inflammatory pain‐induced plasticity.

#### Cerebellar parallel fibre‐stellate cell synapses

Cerebellar stellate cells (SCs) normally express CP‐AMPARs at their parallel fibre inputs from granule cell (GCs). High frequency presynaptic activity triggers a rapid switch from CP‐AMPARs to GluA2‐containing CI‐AMPARs causing a postsynaptic form of LTD (Liu & Cull‐Candy, 2000; Gardner *et al*. 2005), a change that can also be generated by activating mGluR1 receptors with an applied agonist. Furthermore, experiments using mGluR1 blockers have demonstrated that tonic mGluR1 activation normally exerts a suppressive effect on CP‐AMPAR expression at these synapses (Kelly *et al*. 2009). This has provided a useful model for comparison with other forms of CP‐AMPAR plasticity, including those that are detrimental. Indeed, SC plasticity shows some intriguing parallels to that at synapses in the VTA where similar changes occur developmentally, even though the AMPAR subunits and auxiliary proteins are likely to differ (Mameli *et al*. 2011; Loweth *et al*. 2013).

Experiments on SC synapses have also allowed a direct test of whether γ2 is required for CP‐AMPARs to localize at synapses in the cerebellum. GCs and SCs each express two TARPs, γ2 and ‐7. In the *stargazer* mouse, where SCs express only TARP γ7, parallel fibre stimulation still evokes EPSCs (Bats *et al*. 2012). However, these were shown to be readily blocked by PhTx‐433, indicating that the current was carried by CP‐AMPARs. Although the CP‐AMPARs could still localise at synapses in the absence of γ2, they were strongly inwardly rectifying due to block by intracellular polyamines. This, together with a low single‐channel conductance and slow kinetics, suggested the synaptic CP‐AMPARs were TARPless, while the extrasynaptic ones had characteristics of TARPed CP‐AMPARs. γ7 is the only remaining TARP in *stargazer* SCs, suggesting that although synaptic receptors are likely to be TARPless, those in the extrasynaptic membrane are associated with γ7. A different study also observed a dramatic increase in rectification of SC EPSCs (and of extrasynaptic AMPARs) in *stargazer* mice (Jackson & Nicoll, 2011*a*). However, as the authors were unable to detect any increased sensitivity to block by PhTx‐433, they concluded that the increased rectification was unlikely to result from a decrease in AMPAR GluA2 content and hypothesized that it may be attributable to a TARP‐dependent change in receptor gating. Thus, while both these studies suggest a role for γ7 in AMPAR trafficking in *stargazer* SC cells, one concluded it acts non‐selectively (Jackson & Nicoll, 2011*a*) while the other suggests it more likely promotes the presence of synaptic CP‐AMPARs by normally suppressing synaptic expression of CI‐AMPARs while allowing CP‐AMPARs to localize at synapses (Bats *et al*. 2012).

The principle that CP‐AMPARs can localize at central synapses in the absence of γ2 has been tested more generally by examining synapses in cerebellar GCs from *stargazer* mice. These are devoid of miniature EPSCs (Hashimoto *et al*. 1999; Tomita *et al*. 2005), offering an unequivocal experimental scenario. GCs do not normally express CP‐AMPARs, but when GluA2 was knocked down using siRNA mEPSCs unexpectedly reappeared (Studniarczyk *et al*. 2013). These currents were strongly inwardly rectifying suggesting that CP‐AMPARs can indeed localize at synapses in the absence of γ2 and the presence of γ7. Furthermore, transfecting γ7 into wild type GCs (which normally express only CI‐AMPARs) gave rise to inwardly rectifying mEPSCs and whole‐cell currents, supporting the view that γ7 actively enhances CP‐AMPAR expression (Studniarczyk *et al*. 2013). In contrast to these findings, experiments using a knockout mouse have suggested that γ7 does not make a significant contribution to excitatory transmission in either cerebellar SCs or GCs (Yamazaki *et al*. 2015). Thus, at present, the possible role of TARP γ7 in determining features of CP‐AMPAR transmission remains unresolved.

Overall, it is clear that TARPs γ2, ‐5, ‐7 and ‐8 and the atypical auxiliary subunit GSG1L are all potential ‘molecules of interest’ in the regulation of CP‐AMPARs.

### Possible pharmacological and molecular interventions

Changes in the regulation and function of CP‐AMPARs occur in a wide variety neurological conditions and chronic disorders. Therefore, interventions that prevent the aberrant expression, trafficking or targeting of these receptors, or selectively reduce their damaging activation, could prove highly beneficial. A more complete understanding of the molecular mechanisms that underlie CP‐AMPAR regulation is a crucial first step. In this respect an interesting theme has emerged from work that has shown CP‐AMPAR expression to be decreased by the activation of mGluR1s in several different neuron types. Thus, enhancing mGluR1 activation using positive allosteric modulators, which has been suggested as a possible strategy for reversing increased CP‐AMPARs associated with use of cocaine and other addictive drugs (Scheyer *et al*. 2018; Wolf, 2016), could have wide potential.

Epilepsy is another case where insight into the details of CP‐AMPAR regulation has proved useful. One of the notable changes that follows seizures in humans and in mouse models of epilepsy is a dramatic increase in the expression of flip isoforms of GluA1. These not only confer greater glutamate sensetivity than the flop isoforms they replace, but if present in excess could form homomeric CP‐AMPARs. Either of these features might be expected to enhance excitatory synaptic currents. A study by Lykens *et al*. (2017) reported the development of a splice modulating oligonucleotide that decreased GluA1 expression and showed anti‐seizure properties, including reduced post‐seizure hyperexcitability in neonatal mice. Such targeting of specific AMPAR subunit isoforms may have the potential for altering the expression of AMPAR subtypes involved other disease states. Likewise, various molecular approaches, including the use of small interfering peptides (Fosgerau & Hoffmann, 2015), have been used successfully to target protein‐protein interactions and prevent the endocytosis of AMPARs involved in behavioural sensitization models of drug addiction (Dias *et al*. 2012). Small interfering peptides have also been developed to selectively prevent endocytosis of AMPARs containing GluA2 subunits (Lin *et al*. 2016). Clearly, it would be of interest to further develop such approaches to target specific auxiliary subunits that may be involved in CP‐AMPAR delivery.

Perhaps more immediately tractable is the goal of selectively modifying the function rather than expression of CP‐AMPARs. Although the potential of AMPARs as therapeutic targets has been long recognised (see, for example, Bowie, 2008; Rogawski, 2011; Chang *et al*. 2012) there are unique challenges in attempting to pharmacologically interfere with a receptor that is both widespread in the CNS, and fundamental to most aspects of normal brain function. The novel properties of GluA2‐lacking CP‐AMPARs mean that, experimentally at least, it is possible to selectively block their integral ion channel (with existing pharmacological tools such as PhTx‐433, IEM‐1460 and NASPM). These blockers have helped reveal the surprisingly widespread involvement of CP‐AMPARs in various forms of plasticity, including those contributing to neurological disease, and shown diverse therapeutic use in many preclinical studies (e.g. Noh *et al*. 2005; Yennawar *et al*. 2019; Hu *et al*. 2020; Adotevi et al. 2020). As yet, analogues of these drugs have not been successfully developed for wider use, but elegant cryo‐EM work has recently provided invaluable insight into the architecture of the blocker binding site within the pore, and this is likely to provide significant impetus to the further development of small molecule blockers (Twomey *et al*. 2018).

The value of region‐specific therapeutic intervention that can be gained by identifying molecules that target receptor‐associated auxiliary proteins has been considered in recent reviews (Maher *et al*. 2017; Kato & Witkin, 2018). Several such molecules have been described that act as selective antagonists for AMPARs associated with γ8, notably JNJ‐55511118 (Maher *et al*. 2016), LY3130481/CERC‐611 (Gardinier *et al*. 2016; Kato *et al*. 2016) and JNJ‐61432059 (Savall *et al*. 2019). These negative allosteric modulators appear to functionally disrupt the interaction between γ8 and the pore‐forming subunits in the AMPAR assembly and have shown promise as therapeutic approaches for epilepsy (Kato *et al*. 2016; Savall *et al*. 2019) and chronic pain (Knopp *et al*. 2019). The binding of these molecules depends on specific amino acid residues within transmembrane regions of γ8. Introducing the same residues into γ2 and ‐4 confers drug sensitivity on receptors containing these modified TARPs (Maher *et al*. 2016). Of note, a recent study, using molecular dynamics simulations and electrophysiology revealed a conserved moiety among structurally diverse compounds that underlies their interaction within the binding pocket of γ8 (Dohrke *et al*. 2020). The discovery of γ8‐selective drugs, and the growing understanding of how these may act, are exciting developments that could pave the way to the design of antagonists selective for AMPARs containing other TARPs, raising the prospect of tools for region‐specific and CP‐AMPAR subtype‐selective intervention within the CNS.

## Additional information

### Competing interests

The authors have no competing interests and conflict of interests to declare.

### Author contributions

Both authors have read and approved the final version of the manuscript and agree to be accountable for all aspects of the work in ensuring that questions related to the accuracy or any part of the work are appropriately investigated and resolved. All persons designated as authors qualify for authorship, and all those who qualify for authorship are listed.

### Funding

This work is supported by an MRC Programme Grant (MR/T002506/1) to M.F. and S.G.C.C.
